# A Clinical Case of Idiopathic Atrophoderma of Pasini and Pierini With Literature Review

**DOI:** 10.1155/crdm/8886954

**Published:** 2025-03-14

**Authors:** Raksha Pathak, Poshan Neupane, Samir Shrestha

**Affiliations:** ^1^Department of Dermatology and Venereology, Gandaki Medical College, Pokhara, Nepal; ^2^Department of Medicine, Nepal Mediciti Hospital, Lalitpur, Nepal; ^3^Department of Dermatology and Venereology, Rapti Provincial Hospital, Dang, Nepal

**Keywords:** atrophoderma, dermal atrophy, idiopathic, Pasini and Pierini

## Abstract

Atrophoderma of Pasini and Pierini is a rare skin disease that presents with dermal atrophy. Differentiating this condition from morphea remains a challenge. Etiology is unknown, and there is no effective treatment till date. The diagnosis is made through clinicohistopathological correlation.

## 1. Introduction

Atrophoderma of Pasini and Pierini is a rare, cutaneous condition of unknown etiology that causes dermal atrophy [1]. It is sometimes linked to *Borrelia burgdorferi* infection, genetic predisposition, or malignancy [2]. It commonly occurs in females at second to third decades of life and presents as single or multiple, sharply demarcated, hyperpigmented or hypopigmented, atrophic, nonindurated plaques with classic “cliff-drop” borders with no obvious signs of inflammation [3]. We present a case of an 18-year-old male with the diagnosis of idiopathic atrophoderma of Pasini and Pierini (IAPP) along with the review of the literature. The patient presented with six-month history of multiple asymptomatic, depressed, hyperpigmented plaques on the trunk and lower limbs. The diagnosis was made with the clinicohistopathological correlation.

## 2. Case Presentation

An 18-year-old male presented to Dermatology outpatient department, Pokhara Academy of Health Sciences, with history of asymptomatic, hyperpigmented skin lesions over the trunk, and lower limbs for six months. The lesions started as ill-defined discrete ovoid hyperpigmented patches on the trunk which gradually increased in size and number over the months. Thereafter, lesions appeared on bilateral lower limbs. These lesions remained static in terms of size and number for last 2 months. Over the time, these lesions developed central atrophy. There was no history of trauma, insect bites, or new topical or systemic medicines used. Systemic examination did not reveal any abnormalities. There was no significant medical, familial, and travel history.

**Figure 1:**
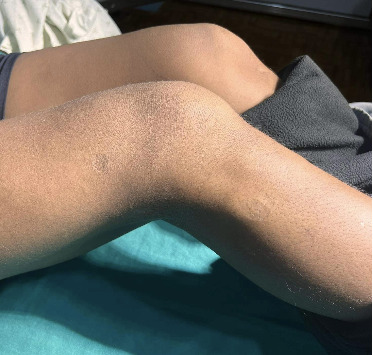
Ovoid well-defined hyperpigmented and atrophic plaques on the lower limbs.

On cutaneous examination, there were multiple, ovoid, well-defined, hyperpigmented, and atrophic plaques on the bilateral distal thighs and proximal legs, distributed asymmetrically, size ranging from 3 × 2 cm to 6 × 4 cm (Figure 1).

**Figure 2:**
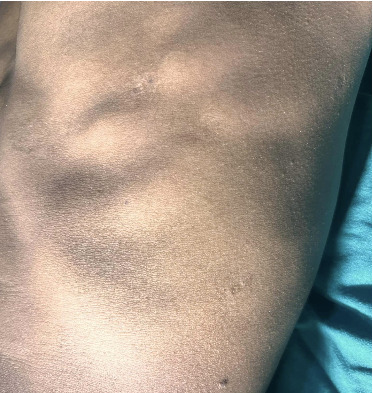
Well-defined, atrophic, ovoid plaques over the trunk with “cliff-drop” borders.

**Figure 3:**
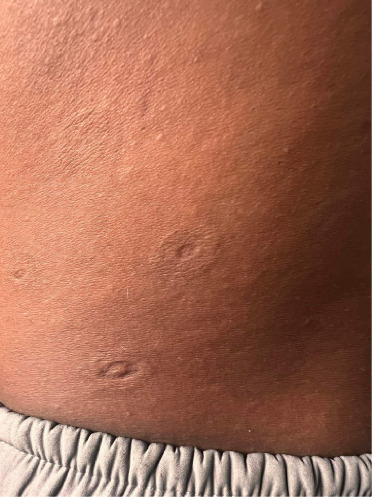
Well-defined, atrophic, ovoid plaques on lower back with “cliff- drop” borders.

On the trunk, there were multiple, well-defined, hyperpigmented ovoid plaques, size ranging from 3 × 2 cm to 5 × 4 cm with a depressed center. The central atrophic area was circular to oval with pathognomonic “cliff-drop” borders (Figures 2 and 3). These lesions were nonindurated, with irregular surface, nontender, and with normal sensation.

**Figure 4:**
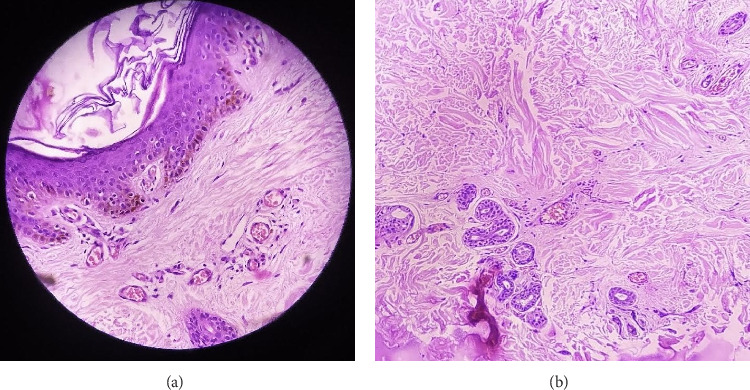
(a) Epidermis showing basket weave hyperkeratosis with basal layer pigmentation. Dermis showing atrophy and dilated blood vessels with mild perivascular inflammatory cell infiltrates comprising of lymphocytes and histiocytes (H and E stain 40x). (b) Dermis with edematous and slightly homogenized collagen bundles with preserved adnexal structures (H and E stain 40x).

Histopathological examination from the lesion on the trunk revealed epidermis with basket weave hyperkeratosis with basal layer pigmentation. There was a decrease in the thickness of the dermis. Upper dermis revealed mildly dilated blood vessels with mild perivascular inflammatory cell infiltrates comprising of lymphocytes and histiocytes. The collagen bundles in mid and deep dermis were edematous and slightly homogenized. Adnexal structures were preserved. No features of malignancy were observed (Figures 4(a) and 4(b)).

Laboratory examination including complete blood count, renal and liver function tests, thyroid function test, blood sugar, lipid profile, rheumatoid factor, antinuclear antibody, syphilis serology test, and anti-Scl-70 antibody test were all within normal limits. The diagnosis of IAPP was made with the clinicohistopathological correlation.

The patient was educated regarding the benign nature of the disease and unavailability of the definitive treatment. Topical calcineurin inhibitor (tacrolimus) was tried for 1 month with no improvement. On follow-up in 1 year, there was no progression of the disease.

## 3. Discussion

Idiopathic atrophoderma was first reported by Pasini in 1923, where he described the condition as “progressive idiopathic atrophoderma” [4]. Later, in 1936, Pierini suggested the potential link between this condition and morphea [5]. Finally, in 1958, Canizares et al. coined the term “idiopathic atrophoderma of Pasini and Pierini (IAPP)” and described this condition as a different entity [6].

IAPP is an uncommon skin condition affecting the dermal collagen organization which results in skin atrophy. The literature shows around 200 reported cases. The disease is commonly seen at second to third decades of life with female to male patient ratio of 6:1 [7]. Cases of congenital atrophoderma have also been reported [8–11].

The etiology of IAPP remains unknown. Some cases of familial atrophoderma of Pasini and Pierini and the concordance between siblings suggest a possible genetic cause [12]. This link is yet to be confirmed. Some authors have linked it to infection with *B. burgdorferi*. In one study, IgG antibodies against *B. burgdorferi* were positive in 53% of patients with IAPP as compared to 14% of controls [13–15]. A neurogenic cause has been suggested because of zosteriform distribution of the lesions in some cases [16]. Some case reports suggest an association with malignancy such as extra medullary plasmacytoma and papillary cancer of the thyroid gland [17, 18].

Whether atrophoderma is a solitary entity or a spectrum of morphea is still debated. IAPP is thought to share a pathogenic pathway with scleroderma [19]. A patient with atrophoderma of Pasini and Pierini was reported to develop progressive systemic sclerosis. In 1995, Paterson included atrophoderma of Pasini and Pierini within the plaque-type morphea. The burnt out lesions of morphea may be clinically and histologically similar to atrophoderma of Pasini and Pierini. There are histological similarities such as collagen homogenization and mild sclerosis [20]. However, the features such as lack of induration and epidermal atrophy, as well as differences in glycosaminoglycan from those seen in morphea, suggest IAPP to be a separate entity [21, 22].

The onset of IAPP is insidious with no obvious signs of inflammation. It presents as single or multiple, well-demarcated, hyperpigmented or hypopigmented, atrophic, nonindurated, irregularly round plaques, with size varying from few millimeters to several centimeters. The lesions develop classic “cliff-drop” borders [23]. They commonly appear on the back and spread to other sites such as chest, abdomen, arms, thighs, legs, and rarely neck and face [15]. The disease progresses slowly over 10–20 years and becomes stable. The existing lesions do not resolve. The lesions are often bilateral. Unilateral zosteriform or blaschkoid pattern has also been reported [16, 24]. They are often asymptomatic but may be associated with pain, pruritus, and paresthesia.

An enzyme-linked immunosorbent assay can be performed to detect antibodies against *B. burgdorferi*. Antinuclear antibodies' titers are positive in some cases [22, 25, 26]. Ultrasonography shows decreased dermal thickness on the affected skin along with an increase in vascularity [25, 27]. The histopathological examination shows a decrease in the thickness of the dermis with the absence of sclerosis. Collagen may show changes such as atrophy, clumping, fragmentation, and hyalinization. Elastic fiber shows reduction and fragmentation. Interstitial edema and mild perivascular infiltrate consisting of lymphocytes and histiocytes are seen in the dermis. The skin appendages are preserved. The overlying epidermis is usually normal [14, 23, 28].

Some studies are carried out to observe the microscopic changes in the skin in IAPP. In a study, multiphoton microscopy found no difference in the content of collagen and elastic tissue but a difference in the organization. The horizontal collagen fibers in the lesion were increased toward the lower portion of the dermis, while elastic fibers depicted greater disorganization within the upper dermis [28]. In another study, direct immunofluorescence testing showed IgM and C3 depositions in the walls of small blood vessels in the papillary dermis, focal fibrinogen in the mid-dermis, and minimal dispersing of IgM cytoids in the basement membrane [1]. In an electron microscopic study, there was the presence of perivascular macrophages and lymphocytes surrounding the vessels [1, 27].

Till date, no definitive treatment has been found for this condition. Topical medications such as corticosteroids, calcineurin inhibitors, and oral medications such as antibiotics, corticosteroids, and hydroxychloroquine have been tried with variable responses [17, 26, 29, 30]. The randomized trials are lacking. Topical steroids and calcineurin inhibitors have showed minimal responses in some cases [26]. Oral prednisolone was found to be ineffective [17]. Doxycycline (200 mg/day) was found to prevent the appearance of new lesions in patients with a positive *B. burgdorferi* antibody titer [13]. A retrospective study of 25 patients treated with either oral penicillin (two million IU/day) or oral tetracycline (500 mg three times daily) for 2-3 weeks showed clinical improvement in 20 patients with no new lesions at follow-up [14]. Hydroxychloroquine has showed good response in cases associated with lupus [26]. Q-switched Alexandrite laser has also been tried with some clinical improvement of hyperpigmented lesions [29]. Topical calcineurin inhibitor (tacrolimus) was tried for 1 month for our patient. Since there was no improvement, the patient refused to continue the treatment.

## 4. Conclusion

IAPP is a rare cutaneous condition of unknown etiopathogenesis. There is dermal collagen disorganization resulting in dermal atrophy. The closest differential diagnosis is morphea, and the relationship between morphea and IAPP is still controversial. There are no definitive treatments till date. Here, we presented a case of an 18-year-old male with the diagnosis of IAPP along with the literature review. Additional research and exploration are required for making concrete conclusions regarding the etiopathogenesis and management of this condition.

## Data Availability

We agree to make the manuscript available to general people and are also ready to provide other necessary data regarding the manuscript in case required.
